# Precursor-Less Coating of Nanoparticles in the Gas Phase

**DOI:** 10.3390/ma8031027

**Published:** 2015-03-11

**Authors:** Tobias V. Pfeiffer, Puneet Kedia, Maria E. Messing, Mario Valvo, Andreas Schmidt-Ott

**Affiliations:** 1 Faculty of Applied Sciences, Delft University of Technology, Julianalaan 136, Delft 2628 BL, The Netherlands; E-Mail: puneetkedia.med@gmail.com; 2 Solid State Physics, Lund University, Box 118, Lund 221 00, Sweden; E-Mail: maria.messing@ftf.lth.se; 3 Department of Chemistry–Ångström Laboratory, Uppsala University, Box 538, Uppsala 751 21 Sweden; E-Mail: mario.valvo@kemi.uu.se

**Keywords:** spark ablation, nanoparticles, coating, gas phase, continuous process

## Abstract

This article introduces a continuous, gas-phase method for depositing thin metallic coatings onto (nano)particles using a type of physical vapor deposition (PVD) at ambient pressure and temperature. An aerosol of core particles is mixed with a metal vapor cloud formed by spark ablation by passing the aerosol through the spark zone using a hollow electrode configuration. The mixing process rapidly quenches the vapor, which condenses onto the core particles at a timescale of several tens of milliseconds in a manner that can be modeled as bimodal coagulation. Gold was deposited onto core nanoparticles consisting of silver or polystyrene latex, and silver was deposited onto gold nanoparticles. The coating morphology depends on the relative surface energies of the core and coating materials, similar to the growth mechanisms known for thin films: a coating made of a substance having a high surface energy typically results in a patchy coverage, while a coating material with a low surface energy will normally “wet” the surface of a core particle. The coated particles remain gas-borne, allowing further processing.

## 1. Introduction

Gas phase production of nanomaterials offers high purity and high throughput. Absolute impurity levels in gas phase systems are orders of magnitudes lower than those in liquids, in part because of convenient purification methods and in part because of the lower specific gravity of gases compared to liquids. Moreover, the solvents and surfactants necessary in liquid phase methods should by themselves be considered impurities. Contrary to a vacuum, the presence of a gaseous carrier allows continuous processing with sequential unit operations. A commonly mentioned drawback of gas phase processing is that one inevitably ends up with an agglomerated product, rather than individual spherical particles. This poses some practical challenges if one wants to functionalize the individual particles, as the coating must be applied before the particles agglomerate further. This requires either lowering the coagulation rate by working at low particle concentrations or working on very short (∼ms) timescales.

When an aerosol of substrate particles of the desired size and concentration is obtained, the coating material can be added using either chemical or physical methods. In chemical vapor deposition (CVD), gaseous precursor molecules are decomposed to form condensing species. Carbon-encapsulated particles can be made by decomposition of hydrocarbons [1]. Inorganic coatings are obtained by decomposition of organometallic complexes, forming oxides such as SiO_2_ [2], Fe_2_O_3_ [3] and Bi_2_O_3_ [4], or noble metals such as Pt [5]. The decomposition requires elevated temperatures, so that typically metal oxides, such as SiO_2_ and TiO_2_, are used as substrate materials. A disadvantage of the CVD approach is that the required precursors tend to be highly reactive and are often toxic.

Physical vapor deposition (PVD) is a cleaner alternative, eliminating both precursor and decomposition products from the process. In PVD, the coating material is first evaporated and subsequently condensed onto the substrate. Evaporation furnaces are suitable if the melting point of the substrate particle is significantly higher than that of the coating material, such as for the deposition of Ga on Au [6] or L-leucine on NaCl or lactose [7]. Organic molecules can be deposited on silver nanoparticles, with lower melting point materials having the tendency to form more uniform coatings [8]. Methods that apply focused heating, such as the axial hot wire used to decorate 10- to 50-nm SiO_2_ particles with 1- to 3-nm Au domains [9], can in theory be used with low melting point substrates.

Spark ablation is a versatile method for the production of nanoparticles ranging from several atoms up to about 20 nm [10]. Each spark is a ∼10 *μ*s electric discharge that rapidly heats the electrode surface to temperatures on the order of 20,000 K, resulting in the ablation of part of the electrode material. The formed vapor rapidly expands into a flow of quenched gas, resulting in quench rates on the order of −10^7^ K·s^−1^. As the gas cools, the vapor condenses to form nanoparticles. Here, we present a simple, flexible and continuous gas phase method for the physical deposition of an arbitrary electrically conductive material onto nanoparticles of any shape, size and composition, at ambient pressure and temperature using spark ablation as a convenient vapor source.

## 2. Theory

**Figure 1.**
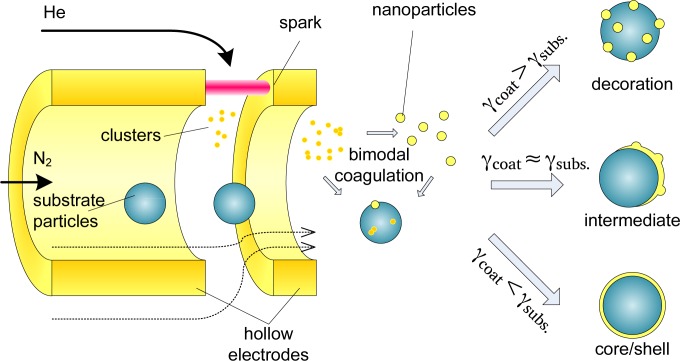
Schematic cross-section of the coating process in a hollow-electrode spark.

In short, substrate particles are mixed with the coating vapor, resulting in a bimodal coagulation process (Figure 1). Homogeneous nucleation of the coating material occurs in parallel, resulting in the growth of coating clusters. However, scavenging [11] results in the preferential deposition of coating vapor and coating clusters on the substrate particles. Substrate-substrate collisions can be neglected due to their lower diffusivity and concentration (appendix A).

Below a certain critical size a deposited vapor cluster is unstable, allowing the cluster to migrate across the substrate surface and enabling epitaxial growth of a coating layer [12]. The deposited clusters rearrange to form a coating according to one of three well-studied growth mechanisms, depending on the relative surface free energies of substrate and adsorbate, *σ*_subs_ and *σ*_ads_, respectively, and their interface energy *σ*_int_. Two-dimensional Frank–van der Merwe (FW) growth occurs if the surface energy of the substrate exceeds that of the adsorbate (*σ*_subs_ > *σ*_ads_ + *σ*_int_), resulting in a smooth, conformal coating. On a low surface energy surface (*σ*_subs_ < *σ*_ads_ + *σ*_int_), a metal-island film grows according to three-dimensional Volmer–Weber (VW) growth. The intermediate case, *σ*_subs_ ≈ *σ*_ads_ + *σ*_int_, can lead to mixed 2D/3D Stranski–Krastanov (SK) growth. These growth methods can also be understood in terms of the physical-chemical interpretations of wetting and non-wetting liquids on surfaces. The above description of film growth is based on thermodynamic equilibrium, valid for single crystalline surfaces, in absence of strain or lattice mismatches. Kinetic effects, such as interdiffusion and strain relief by interfacial alloying, play an equally important role. While thermodynamic arguments alone are clearly insufficient to describe nanoparticles, which have multiple facets that are often strained due to the stress of the large surface curvature, they do present a clear starting point for studying the growth of coatings on unsupported nanoparticles.

Here, we use two metals of similar surface energies: gold (*σ*_ads, Au_ = 1.54 J·m^−2^ [13]) and silver (*σ*_subs, Ag_ = 1.32 J·m^−2^ [13]), as well as gold on gold, to study the intermediate domain. Both Ag and Au have an *fcc* structure, with similar lattice constants of 408.53 pm and Au 407.82 pm, respectively. Both FW- and SK-type growth modes are known for Ag/Au systems, and interfacial alloying is known to occur [14]. Polystyrene (PSL, *σ*_ads,PSL_ = 0.04 J·m^−2^ [15]) nanoparticles were used as low surface energy core particles. Spark ablation is used to evaporate the coating material. This provides three advantages: (1) the coating material is selectively heated, reducing the co-evaporation of contaminants [16]; (2) only a small fraction of the gas is heated, so that the overall gas temperature stays close to room temperature; and (3) the substrate particles can be brought into close proximity of the evaporation source, ensuring that the coating clusters stay below the critical size.

## 3. Experimental Section

**Figure 2.**
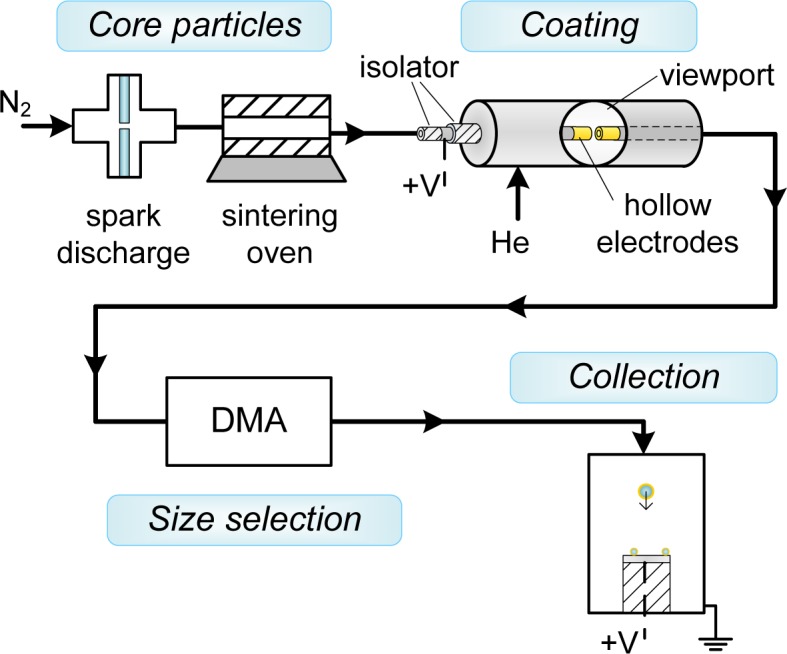
Coating setup comprising a particle source, coating zone, differential mobility analyzer (DMA) and an electrostatic precipitator (ESP).

Au-coated Ag (Au-on-Ag) nanoparticles and Ag-coated Au (Ag-on-Au) nanoparticles were generated in a setup consisting of a particle source for the core particles, a coating zone consisting of a hollow electrode spark, a differential mobility analyzer to isolate the coated particles and a collection zone (Figure 2). The setup was operated at slightly above atmospheric pressure. Ag and Au core particles were formed by spark ablation [10] (20 nF, 30 mA, 1.1 kV–1.2 kV) in a flow of 1.6 L·min^−1^ N_2_ (room temperature, 1 atm), followed by reshaping of the formed agglomerates to ∼40-nm spheroids using a sintering furnace (42 cm hot zone, *T*_furnace_ ∼90% of the melting point) [17]. Diluted suspensions of polystyrene-latex (PSL) spheres (80 nm ± 18%, Duke Scientific, 5008A, Palo Alto, CA, USA) in demineralized water were nebulized in an atomizer (TSI 3076) and dried in a diffusion drier loaded with silica gel [18]. The combination of the atomizer and the diffusion drier replaces the first spark and the sintering furnace in Figure 2, and no differential mobility analyzer (DMA) was used with PSL cores.

The concentration of core particles measured with a condensation nucleation particle counter (TSI 3776, TSI Inc., Shoreview, MN, USA) downstream of the size selection step ranged from 0.5·10^13^ m^−3^ to 1.5·10^13^ m^−3^. All flow lines consisted of 6-mm stainless steel tubing with Swagelok fittings, and sufficient length (∼30 cm) was provided to ensure the core particle aerosol had cooled to room temperature before entering the coating zone.

The coating zone (schematically represented in Figure 1) consisted of two axially-aligned hollow electrodes (Ag or Au, inner diameter 4 mm, outer diameter 6 mm) spaced ∼0.5 mm apart, contained within a stainless steel housing with NW35CF flanges. The coating metal was ablated using low energy sparks (2 nF, 15 mA, 0.3 kV (Ag), 0.7 kV (Au)) to avoid the formation of large particles. A sheath flow of the low breakdown strength gas helium (2 L·min^−1^) was used to both allow a lower spark energy in the gap [10] and to introduce a forced mixing of the metal vapor with the core particles, taking advantage of the high diffusion coefficient in He. The vapor condensed on the core particles, until the coating process was stopped by removing the fine fraction in the aerosol using electrical mobility separation in a DMA [19] with a 2:1 sheath-to-aerosol ratio. This was possible due to the self-charging that occurs in spark discharge [10]. Finally, the coated particles were deposited during 20 min on various substrates (TEM grids, Si wafer, glass) using an electrostatic precipitator (ESP) before further characterization.

Transmission electron microscopy (TEM) and energy dispersive spectroscopy (EDX) were performed on a JEOL 300F microscope (JEOL, Akishima, Tokyo, Japan) with a field emission gun (FEG) at 300 kV. Additional TEM was performed using an FEI monochromated Tecnai 200STEM-FEG (FEI, Hillsboro, OR, USA) at 200 kV and a JEOL JEM 1400 at 100 kV. Raman spectra were recorded by a inVia Raman microscope (Renishaw, Wotton-under-Edge, UK) using laser excitation wavelengths of 532 and 633 nm and applying a nominal power of 0.5 and 0.05 mW, respectively. The lens used in the optical microscope during the measurements provided a 50× magnification, in order to easily focus the laser beam onto the mesh wires of the various coated TEM grids. All of the spectra were calibrated on the basis of the peak at 520.6 cm^−1^ obtained from a preceding measurement on a (100)Si wafer. Twenty cumulative acquisitions were run in order to improve the S/N ratio in the ultimate spectra, which had a measuring time of 20 s for all of the specimens.

## 4. Results and Discussion

### 4.1. Coagulation Model

**Figure 3.**
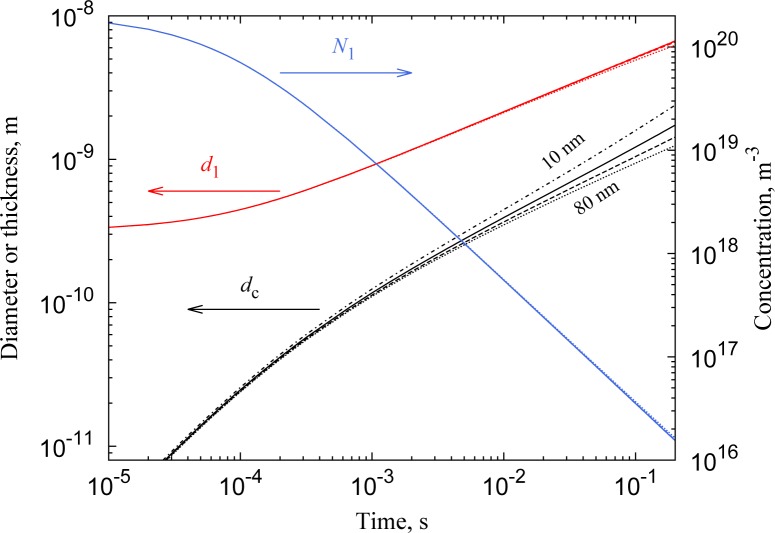
Simulation results for cluster concentration (*N*_1_, blue), cluster size (*d*_1_, red) and coating thickness (*d_c_*, black), using core particle sizes of 10 nm (solid), 20 nm (dash), 40 nm (dash-dot) and 80 nm (dots).

The coating process can be described adequately by a simple bimodal coagulation model (appendix A). Large particles (the cores) are known to preferentially scavenge smaller ones (the clusters) [18], and for 10–100 nm nanoparticles, the coagulation kernel *K* of cluster-core collisions exceeds the cluster-cluster coagulation kernel by up to four orders of magnitude (Equation 2). Simulations performed for core particle concentrations of 1.5·10^13^ m^−3^ and core particle sizes of 10, 20, 40 and 80 nm show that the coating process occurs on a sub-second timescale (Figure 3). The rate at which the available clusters *N*_1_ disappear and the size *d*_1_ to which they grow are weakly dependent on the core size *d*_2_. The rate at which clusters deposit on the core particles scales with *d*_2_^2^, *i.e.*, the available surface area. The coating thickness *d_c_* grows faster for smaller particles despite this constant flux, because they need less mass (∝ *d*^3^) to achieve the same growth in diameter.

While collisions between core particles can be neglected, a non-negligible growth of the clusters occurs. This is because the cluster concentration is much larger than that of the core particles, compensating for the lower *K* of cluster-cluster collisions. Smooth coatings can only be obtained when the deposited clusters have not yet reached their critical size. Below ∼3 nm, both Ag and Au nanoparticles are essentially liquid-like at room temperature [20], and we assume that this size corresponds to the critical size. To allow the growth of smooth layers, we stop the coating process by filtering out the clusters when they have reached the critical size after a residence time of 40 ms (Figure 3). At this stage, the coating thickness has grown to 0.6–0.8 nm, while the cluster size *d*_1_ has grown to 3.5–3.7 nm.

### 4.2. Au on Au

**Figure 4.**
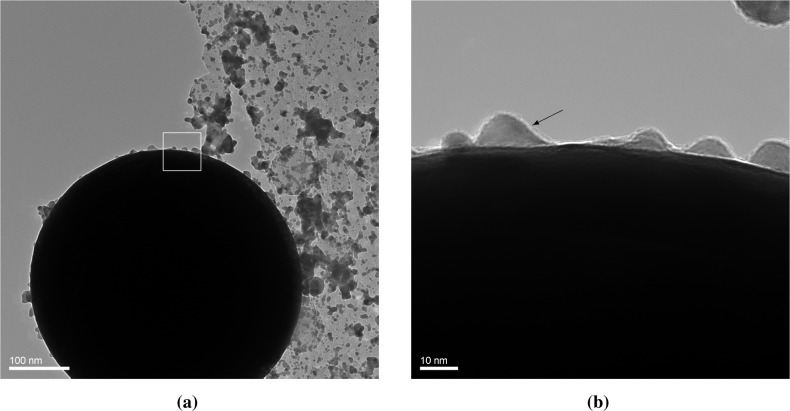
Au clusters deposited on an Au microsphere. (**a**) Au microsphere with Au clusters; (**b**) detail of the microsphere, with Au [111] lattice fringes indicated by the arrow. Scale bars are 100 nm and 10 nm, respectively.

Figure 4a shows a 0.45-μm sphere of Au, collected during deposition of Au on PSL with a high spark energy. The microsphere is a solidified droplet. Such droplets are occasionally ejected in spark ablation apart from the vapor [10]. They are usually undesired, but here this event gives us the opportunity to observe the coating of Au particles with Au nanoparticles and clusters. Vapor and clusters rapidly attach to the large gold cores in the vicinity of the spark. As we do not separate the coating particles from the core particles in this experiment, we allow the coating particles to grow to a size around 10 nm, and they can still attach to the core particles at that stage.

The TEM micrographs (Figure 4a) show dome-like structures that have evidently been formed by the attachment of particles between 5 and 10 nm in diameter. As we know that in the vicinity of the spark a rapid attachment of much smaller particles occurs, the absence of smaller domes tells us that these smaller particles form a smooth coating, not leaving any visible traces for the case of Au on Au. This observation is in agreement with the fact that small particles are liquid-like below a certain size [20] as a consequence of reduced binding energies of surface atoms due to the surface curvature. In independent experiments, we have frequently observed that Au particles exhibit complete coalescence into a spheroidal shape if they are smaller than 5 nm [21]. The reduction of surface curvature by the dome formation or wetting process leads to solidification. We conclude that for the case of equal surface free energies of the core and the coating particles, smooth coatings can be formed if the latter are smaller than this liquefaction size. In contrast, island structures form if larger particles are deposited. The complete coalescence of <5 nm Au particles is confirmed by the single, round clusters deposited on the holey carbon film of the TEM grid having a modal size of 2–4 nm, while non-spherical agglomerates start appearing above 3 nm. The wetting droplets and the single round clusters validate the assumption of fast coalescence of <3 nm particles, *i.e.*, liquid-like particles, allowing the clusters in this size range to be modeled as simple spheres.

### 4.3. Au Clusters on PSL Cores

**Figure 5.**
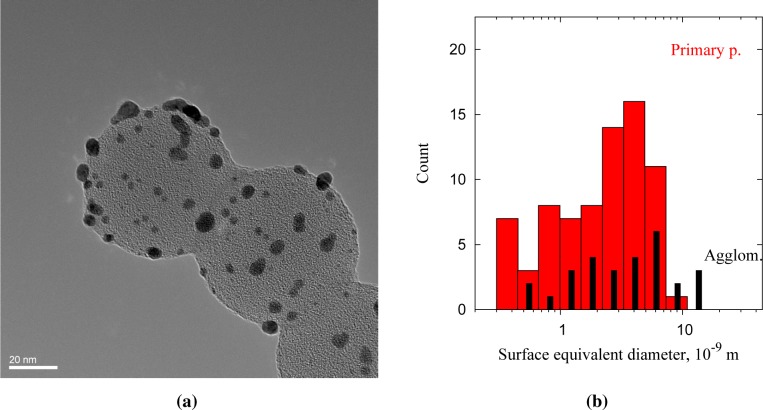
Au clusters deposited on polystyrene (PSL) nanospheres. (**a**) TEM micrograph, 20-nm scale bar; and (**b**) size distribution of primary (round) and agglomerated cluster domains, 105 in total.

The Au clusters (dark) deposited on the PSL core particles (bright) decorate the surface of the spheres (Figure 5a). The PSL spheres had diameters of 66 ± 7 nm and were deposited as singlets, doublets and multiplets. The size of the clusters ranges from 1 to 10 nm, and the dark spots are confirmed to be gold from lattice spacings of 0.23–0.24 nm (Au [111]). Figure 5b shows the size distribution of 105 clusters deposited on PSL spheres, with circular (primary) particles and non-circular particles (agglomerates) shown separately. The shape of surface equivalent diameters below 1.3 nm cannot be resolved sufficiently to distinguish spherical particles from agglomerates, but non-circular particles are categorized as agglomerates for consistency. Only clusters overhanging the holes in the carbon film are taken into account, to avoid counting clusters that arrived on the TEM grid independently of the PSL cores. The size distribution normalized to a single PSL sphere corresponds to a deposited volume of 4.8·10^3^–6.9·10^3^ nm^3^, or 1·10^5^ Au atoms per PSL particle. The model for 80-nm PSL spheres overpredicts at 7·10^5^ atoms per particle, although the modal size of the clusters, 2–5 nm (Figure 4b), fits rather well. The discrepancy in deposited gold can be explained by the presence of dimer and oligomer PSL particles, resulting in a larger PSL surface area at the measured particle number concentration.

### 4.4. Au Clusters on Ag Cores

**Figure 6.**
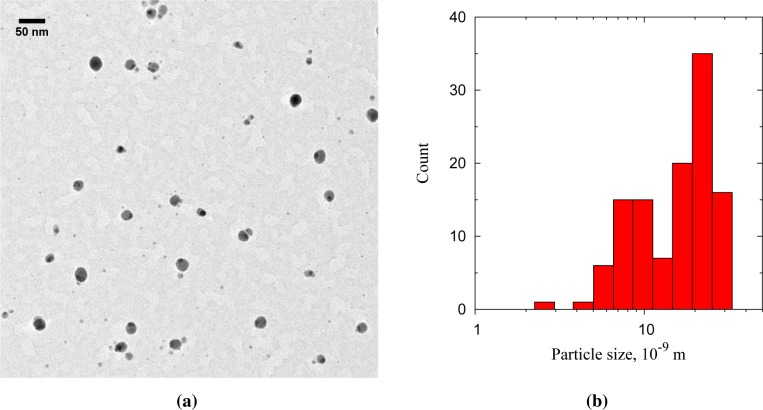
(**a**) TEM micrograph of Au-on-Ag particles with s 50-nm scale bar; (**b**) particle size distribution of 116 particles.

**Figure 7.**
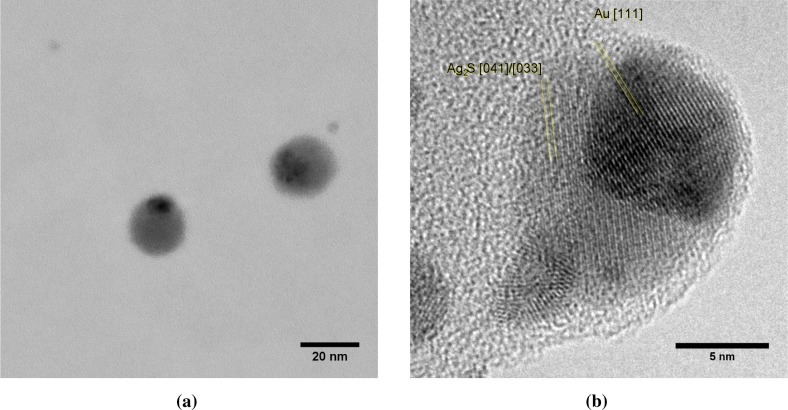
TEM micrograph of Au-on-Ag particles. (**a**) Two patchy particles and two clusters; (**b**) HRTEM image with lattice fringes of Ag_2_S (0.334 nm) and Au (0.231 nm). Note the different scale bars of 20 nm in (**a**) and 5 nm in (**b**).

The Au-on-Ag particles appear as light cores with a single dark cap (Figures 6 and 7a). The Au-on-Ag particles size has a broad distribution, with a modal size of 21 nm (Figure 6b). The broadness of the size distribution has three causes: (1) the core particle source was not optimized for monodispersity; (2) the DMA was operated with a broad transfer function to allow high transmission; and (3) doublets of smaller particles were also collected. These doublets either formed between the sintering oven and the cluster source or downstream of the cluster source. Because the residence time prior to the cluster source (∼400 ms) was much larger than that downstream of the source (40 ms), most of these doublets were likely formed before the cluster source. From the limited number of doublets observed, we conclude that core-core particle collisions can indeed be neglected in the coating process. Starting with a monodisperse aerosol source, e.g., by size selection with an additional DMA, is thus expected to result in equally monodisperse bimetallic particles.

Each core particle had a dark patch of at least 7 nm in diameter (Figure 7). The majority of the cores has exactly one spot, although some non-spherical particles with two dark patches were also observed. Lattice spacings of the dark patches match Au (Figure 7b), but due to the similar lattice constants, could also originate from Ag or an Ag-Au alloy. The light zones do not show lattice spacings consistent with Ag. Instead, lattice spacings of 0.167 nm (Figure 7b) are attributed to Ag_2_S, a well-known silver tarnish that forms upon exposure to ambient air [22,23]. The relative abundances of Au, Ag and S in energy dispersive X-ray spectroscopy (EDX) are consistent with the light zones being sulfidated Ag and the dark zones being Au. Ten individual particles had a Au content of 17% ± 13% on a metal basis, with the remainder being Ag. This is equivalent to a conformal coating thickness of 1.1 to 1.3 nm, which is somewhat larger than the 0.6 to 0.8 nm obtained from the coagulation model.

Au clusters with a mean diameter of 3.6 nm were observed in TEM micrographs alongside Au-on-Ag particles (Figure 7a). The high diffusivity of the 3.6-nm clusters allowed a fraction of these clusters to pass through the DMA. The size of these clusters is again consistent with the cluster size obtained from the simulations. In particles where the dark spot is on the edge of the particle, the Au appears to envelop the Ag core (e.g., Figure 7a) or to be partially embedded. This is consistent with the mechanism of Au forming a patch covering the lighter Ag core, rather than a doublet of odd-sized particles aligned parallel to the electron beam. Burrowing of the Au domain into the Ag particle by interdiffusion, such as observed for thin films [14], could neither be confirmed nor excluded.

### 4.5. Ag Clusters on Au Cores

**Figure 8.**
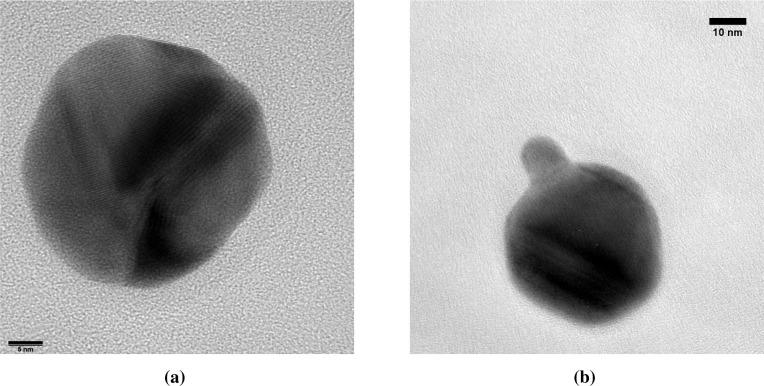
TEM micrographs of Ag-on-Au nanoparticles, (**a**) 5-nm scale bar; (**b**) 10-nm scale bar.

The Ag-on-Au particles have a broad distribution of particle sizes ranging from 27 to 130 nm and consist of a mixture of sintered spheres (Figure 8a) and agglomerates (Figure 8b). The extent of neck formation implies that the agglomerates formed by coagulation in the sintering oven, as a result of a higher initial Au core particle concentration. The particles are identified as gold from EDX and Fourier transform lattice spacings of 0.23–0.24 nm (Au [111]). Ag could not be detected by EDX on 30-nm particles, where a 1-nm coating would correspond to molar content of ∼10% Ag. No distinct layer of any Ag compound was visible on any of the Ag-on-Au particles under HRTEM. Due to their similar lattice spacings, Ag and Au cannot be distinguished within the resolution of the lattice spacing measurements. The 0.6 to 1.0 nm coating thickness predicted by the coagulation model corresponds to 3–5 monolayers. Even in absence of interfacial alloying [14], the high density of the gold core combined with the similar lattice parameters of the two metals would make it difficult to see a silver shell of only several monolayers thick.

**Figure 9.**
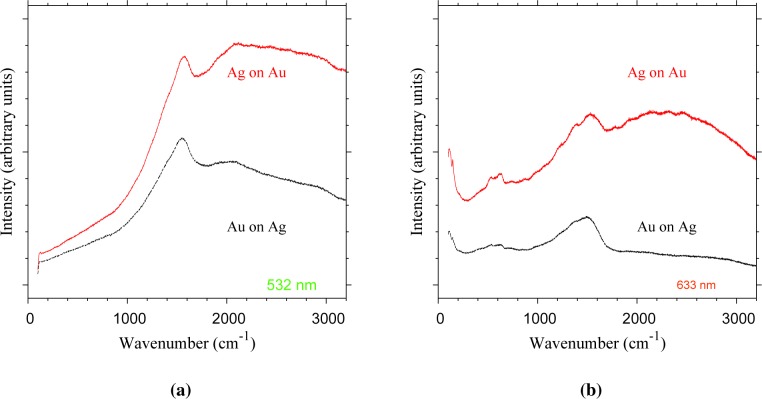
Raman spectra of Au-on-Ag and Ag-on-Au nanoparticles for excitation at (**a**) 532 nm and (**b**) 633 nm.

To test for Ag-based tarnish compounds, Raman spectroscopy was used to probe the particle surface of both Ag-on-Au and Au-on-Ag nanoparticles (Figure 9). The assumption is that surface-enhanced Raman scattering (SERS) [24] occurs for, e.g., Ag_2_S domains sitting on the noble metal core particles undergoing their characteristic surface plasmon resonance. The particles of both configurations show strong Raman signals under both excitation wavelengths (Figure 9). Under 532-nm excitation, a strong mode appears at 1575 cm^−1^ for Au-on-Ag particles and at 1540 cm^−1^ for Au-on-Ag particles (Figure 9a). Under excitation by a 633-nm laser, vibrational modes appear at 111, 146, 539, 635, 1243, ∼1396 and 1529–1550 cm^−1^ for Ag-on-Au particles (Figure 9b). The stronger of these features also appears for Au-on-Ag particles at lower wavenumbers with a shift of about 5 cm^−1^. The Ag-on-Au particles show a broad band in the 1800–3000 cm^−1^ range at both wavelengths, which likely is the result of fluorescence. The strong Raman signals indicate that SERS indeed occurs. However, it should be noted that most of these vibrational modes also appear weakly during the analysis of blank Cu TEM grids, thus indicating that the enhanced signals in Figure 9 likely originate from contaminants on the TEM grids. Therefore, the presence of Ag tarnish compounds cannot be concluded from these measurements. It is possible that insufficient Ag was deposited to be detected by our available methods. Ag has a lower ablation rate than Au [10], which would lead to a relatively low Ag concentration for the Ag-on-Au nanoparticles.

### 4.6. Coating Morphology

The different morphologies of the Au coatings show that the deposited clusters can diffuse across the particle surface under ambient conditions, provided they are sufficiently small. The islands on PSL (*σ*_ads_ ≫ *σ*_subs_) and the singular domains on Ag (*σ*_ads_ ≈ *σ*_subs_) are consistent with physical (epitaxial growth) or chemical (wettability) interpretations. The presence of <1-nm clusters on PSL (Figure 5) shows that surface aggregation is limited by slow surface diffusion of Au clusters on PSL. This is consistent with observations that noble metal clusters tend to partially embed themselves into polymer surfaces [25].

On Ag, the Au clusters can migrate more freely across the particle surface, resulting in the presence of a single Ag domain on each particle. Because each particle has exactly one or two Au domains, but never none, their formation cannot be explained by the deposition of a single large cluster, such as observed for Au-on-Au (Figure 4b), as that would imply preferred coagulation by similarly-sized particles over differently-sized particles. The domains appear to be smooth and contiguous, indicating that as the surface domains grow, they have sufficient time to reorganize between subsequent cluster-cluster collisions. Molecular dynamics simulations of the sintering of two ∼2-nm Au particles show that restructuring of the agglomerates occurs on a timescale of 10^−9^ s, with necks disappearing within 0.3 ns [26]. The coagulation simulations show that the deposition flux *F* drops from 10^22^ to 10^20^ m^−2^·s^−1^ during the coating process. For a 20-nm particle, this means that the mean time between clusters depositing on the particle surface is 10^−7^ s to 10^−5^ s, giving ample time for restructuring in the case where a depositing particle directly deposits onto an existing coating domain. The mean island distance *l* can be estimated from Villain’s model as *l* ≈ (*D/F*)^1/6^, with *D* and *F* scaled to the crystal unit cell [12]. *D* is the tracer diffusion coefficient, which we assume to be similar to that of Au on Au for Au on Ag, *i.e.*, 10^−8^ m^2^ s^−1^ [26]. The calculated mean island distance for Au on Ag for the conditions in these experiments is 15 nm, consistent with the formation of a single island on 20-nm particles.

## 5. Conclusions

In summary, thin metal coatings were deposited on nanoparticle aerosols by an atmospheric PVD process. No precursors, solvents or surfactants are required, simply a spark to locally heat the target electrodes, making it a clean and flexible method for coating nanoparticles. The geometry of the coating system allows for short residence times, which keeps the coating clusters small enough to allow the films to restructure upon deposition. The final morphology of the coatings is understood through the relative surface energies of the two materials, somewhat similar to thin film growth mechanisms. While Au formed a multitude of small clusters when deposited on low surface energy PSL particles, it formed a single smooth patch on Ag. With the analytical methods at our disposal, we were not able to confirm conformal coating of Ag on Au. However, the fact that our model gives a realistic measure of the flux of coating material to the core particles for Au on Ag and Au on PSL gives us certainty that Ag coating of Au must have taken place. The results on Au on Au, where only deposition of particles too large to be liquid-like led to island-growth, indicates that cluster attachment near the spark gap must lead to a smooth coating for suitable surface energies.

Despite several simplifying assumptions, the bimodal coagulation model gives a reasonable measure for both the Au cluster size and the amount of Au deposited on the substrate particles. This makes it possible to tune the coating process to involve only mobile clusters. According to the model calculations, a coating of 1–2-nm thickness can be deposited in a single pass through the coating zone, before the clusters grow bigger than our critical size of 3 nm. Better accuracy can be achieved by building on the more mature models developed in aerosol CVD, which include polydispersity, and more refined approximations for the transition regime. The continuous nature of the process in principle allows for the growth of multiple layers (each of a different composition, if so desired) by placing several hollow sparks and DMAs in series.

## A. Bimodal Coagulation Model

We describe the coating as a bimodal Smoluchowski-type coalescence model [18], where the rate of change in particle concentration *N* is related to the coagulation kernel *K*.

(1) $$\frac{dN}{dt}=-KN^{2}$$

The coagulation kernel between particles of size *i* and *j*, *K_ij_*, is calculated using Dahneke’s approximation for the transition regime and free molecular regime [27,28]:

(2) $$K_{ij}=K_{\text{continuum}}\frac{2+\kappa }{2+\kappa (2+\kappa )},$$

where *κ* is the ratio of coagulation kernels in the continuum and free molecular regimes:

(3) $$\kappa =\frac{K_{\text{continuum}}}{K_{\text{free molecular}}}=\frac{\pi (d_{i}+d_{j})(D_{i}+D_{j})}{\frac{\pi }{4}{\left(d_{i}+d_{j}\right)}^{2}\sqrt{{\bar{\upsilon }}_{i}^{2}+{\bar{\upsilon }}_{j}^{2}}}$$

The diffusion coefficient *D* is calculated using Allen and Raabe’s approximation for the slip correction factor [29]. Because *d* increases with decreasing *D*, the collision kernel for particles of unequal size is much greater than that of equally-sized particles, resulting in so-called scavenging of small particles by large particles [11].

Coagulation is tracked using the concentration of particles, *N_i_*, and atoms *n_i_*. The clusters that form the coating are labeled with index 1, substrate particles with index 2. Index *c* refers to clusters deposited onto substrate particles. Collisions involving a cluster result in the consumption of one cluster, *i.e.*, a cluster and a substrate particle yield one substrate particle. Collisions are assumed to lead to immediate sintering, and particle diameters are calculated from the volume of the deposited coating, assuming conformal coating. Diffusion losses d*N_w,_*_1_/d*t* are estimated using Hinds’ approximation of Gormley and Kennedy’s equations [18,30]. The four resulting differential equations:

(4a) $$\frac{dN_{1}}{dt}=\;-K_{11}N_{1}^{2}\;-2K_{1,2}N_{1}N_{2}-\frac{dN_{w,1}}{dt}$$

(4b) $$\frac{dN_{2}}{dt}=\;-K_{22}N_{2}^{2}\;-\;\frac{dN_{w,2}}{dt}$$

(4c) dn1dt=−2n1N1K12N1N2−n1N1dNw,1dt=−2K12n1N2−n1N1dNw,1dt

(4d) dncdt=2n1N1K12N1N2=2K12n1N2

are solved in MATLAB (MathWorks).
